# Changing the Paradigm in Prognostic Breast Cancer Testing Based on Extracellular Vesicles

**Published:** 2023-06-22

**Authors:** Malgorzata A. Witek, Steven A. Soper, Andrew K. Godwin

**Affiliations:** 1 Department of Chemistry, The University of Kansas, Lawrence, KS 66047, USA; 2 Center of Bio-Modular Multiscale Systems for Precision Medicine (CBM2), The University of Kansas, Lawrence, KS 66047, USA; 3 Kansas Institute for Precision Medicine, University of Kansas Medical Center, Kansas City, KS 66160, USA; 4 Department of Cancer Biology, The University of Kansas Medical Center, Cancer Center, Kansas City, KS 66160, USA; 5 Department of Pathology and Laboratory Medicine, University of Kansas Medical Center, Kansas City, KS 66160, USA

## ABOUT THE STUDY

Women with early-stage breast cancer have several options for genomic tests including Oncotype DX^®^, MammaPrint^®^, and Prosigna^®^ that can estimate risk of distant recurrence and help determine whether they will benefit from adjuvant chemotherapy, but these tests depend on access to tumor tissue samples ^[1-3]^. Our recently published work on “Assessing Breast Cancer Molecular Subtypes Using Extracellular Vesicles’ mRNA” was driven by a desire to develop a liquid-based biopsy approach to estimate the risk of distant recurrence for breast cancer patients receiving endocrine therapy ^[4]^.

The clinical relevance in therapeutic stratifications of different molecular subtypes of breast cancer has been demonstrated ^[5]^. Molecular subtyping in breast cancer looks beyond the common prognostic clinical-pathological parameters (e.g., age, tumor size, presence of node metastases and histological grade) and receptor status (i.e., progesterone, estrogen, and HER2) to make sure breast cancer patients receive proper treatment. One such test is the Prosigna^®^ Breast Cancer Prognostic Gene Signature Assay (formerly called the PAM50 test). Prosigna^®^ is used to estimate the risk of distant recurrence for postmenopausal women within 10 years of diagnosis of early-stage, hormone-receptor-positive disease with up to three positive lymph nodes after 5 years of hormonal therapy.

Our study focused on molecular profiling and subtyping of breast cancer patients using a minimally invasive approach. We focused our attention specifically on Extracellular Vesicles (EVs) and their molecular cargo (i.e., exo-mRNA) that can be secured from a simple blood draw. The premise of our research was that EVs carry RNA cargo believed to be associated with the cell-of-origin and thus, have the potential to serve as a liquid biopsy marker for supplying molecular information to identify characteristics of the disease (i.e., precision medicine). EVs were affinity selected by targeting epithelial and mesenchymal surface markers and the harvested exo-mRNA cargo was analyzed using the PAM50^®^ gene panel on the Nanostring nCounter. We also developed a new analysis algorithm (exo- PAM50), which aided in the examination of the exo-mRNA data. Our initial results were very encouraging and suggested that the quantitation of PAM50-based exo-mRNA transcripts collected from both epithelial and mesenchymal EVs could generate profiles with high concordance to tumor tissue-based testing.

Additional studies are required to further validate our exo-mRNA-based PAM50 assay and potentially other clinical approved prognostic tests in breast cancer patients; however, in our work we have demonstrated the feasibility of using novel methods to efficiently isolate exo-biomarkers and their analysis with the validated technologies or clinical lab-developed tests. Liquid biopsy assays will be important in the future as they can be used in the stratification and subtyping of other epithelial cancers. For example, molecular subtypes have been identified for other epithelial malignancies such as pancreatic, colorectal, prostate, bladder, and sub-classification of triple-negative breast cancers ^[6-10]^.

Extracellular Vesicles (EVs) have the potential to function as a minimally invasive liquid biopsy marker for delivering molecular information to assist treatment decisions (i.e., precision medicine) since they carry RNA cargo that is thought to be connected with the cell-of-origin. We describe the high-volume production-capable affinity isolation of EV subpopulations using monoclonal antibodies mounted to the surface of a microfluidic chip made of plastic. The efficacy of the EV-Microfluidic Affinity Purification (EV-MAP) chip was established in a proof-of-concept application to give molecular subtyping data for breast cancer patients by isolating EVs derived from two-orthogonal cell types.
